# Identification of QTL underlying physiological and morphological traits of flag leaf in barley

**DOI:** 10.1186/s12863-015-0187-y

**Published:** 2015-03-20

**Authors:** Lipan Liu, Genlou Sun, Xifeng Ren, Chengdao Li, Dongfa Sun

**Affiliations:** College of Plant Science and Technology, Huazhong Agricultural University, Wuhan, 430070 China; Biology Department, Saint Mary’s University, 923 Robie Street, Halifax, NS B3H 3C3 Canada; Department of Agriculture and Food/Agricultural Research Western Australia, 3 Baron-Hay Court, South Perth, WA 6155 Australia; Hubei Collaborative Innovation Center for Grain Industry, Wuhan, 430070 China

**Keywords:** Barley, Net photosynthetic rate, Stomatal conductance, Flag leaf area, Flag leaf length, Flag leaf width, Relative chlorophyll content, Leaf nitrogen concentration

## Abstract

**Background:**

Physiological and morphological traits of flag leaf play important roles in determining crop grain yield and biomass. In order to understand genetic basis controlling physiological and morphological traits of flag leaf, a double haploid (DH) population derived from the cross of Huaai 11 × Huadamai 6 was used to detect quantitative trait locus (QTL) underlying 7 physiological and 3 morphological traits at the pre-filling stage in year 2012 and 2013.

**Results:**

Total of 38 QTLs distributed on chromosome 1H, 2H, 3H, 4H, 6H and 7H were detected, and explained 6.53% - 31.29% phenotypic variation. The QTLs flanked by marker Bmag829 and GBM1218 on chromosome 2H were associated with net photosynthetic rate (Pn), stomatal conductance (Gs), flag leaf area (LA), flag leaf length (FLL), flag leaf width (FLW), relative chlorophyll content (SPD) and leaf nitrogen concentration (LNC).

**Conclusion:**

Two QTL cluster regions associated with physiological and morphological traits, one each on the chromosome 2H and 7H, were observed. The two markers (Bmag829 and GBM1218) may be useful for marker assisted selection (MAS) in barley breeding.

## Background

Barley (*Hordeum vulgare* L.) is the fourth cereal crop in world production [1]. High yield is always one of the important barley breeding aims [2]. However, grain yield was controlled by complex biochemical and physiological processes, and closely related to physiological and morphological traits [3-7]. The top three leaves on a stem, especially the flag leaf, absorb most irradiation light, and were the primary source of carbohydrate production [8]. In barley, importance of flag leaf on increasing grain yield has widely been studied [6,7,9]. However, previous studies have mainly focused on either morphological traits [10-12] or physiological traits of flag leaf [13-18] determining grain yield. Few QTLs associated with these traits have been applied to barley breeding due to complicated measurement procedure, inconsistency and dynamic process of physiological and morphological traits in barley developmental stage. Thus, comprehensive understanding the role of physiological and morphological traits of flag leaf on yield will provide a new insight in crop growth and development. Meanwhile, application of molecular marker and genetic map made it possible to map the region controlling quantitative traits [11,19,20].

Increasing photosynthetic capacity of leaf is one of the most important approaches to increase crop biomass [21]. It was estimated that leaf photosynthesis contributing 30% biomass [2]. Photosynthesis is an essential process to maintain crop growth and development. Photosynthetic capacity during reproductive stage is positively correlated with crop yield [22]. Four main physiological parameters: net photosynthetic rate, stomatal conductance, intercellular CO_2_ concentration and transpiration rate, have been used to evaluate photosynthetic capacity. Teng et al. [2] reported that net photosynthetic rate in rice was controlled by multiple genes. In barley, QTL underlying net photosynthetic rate has been analyzed in two DH populations [18]. According to Jiang et al. [23], stomatal conductance significantly affected net photosynthetic rate, and is a key parameter to assess limitation of photosynthesis in barley. Rybiński et al. [24] found significant linear relationship between transpiration rate and net photosynthetic rate in different irradiated times under laser light. However, the QTLs underlying stomatal conductance, intercellular CO_2_ concentration and transpiration rate have not been reported in barley.

Chlorophyll absorbs light energy and converts it into chemical energy. Maintaining higher level of chlorophyll content in leaf is one of the strategies for increasing photosynthesis and crop production [14]. The structure and function of chloroplasts determine photosynthetic activity [25]. Von Kroff et al. [26] reported a positive correlation between relative chlorophyll fluorescence in leaf and grain yield. The chlorophyll content was suggested as a reliable indicator for evaluating metabolic balance between photosynthesis and yield performance [27]. Recently, chlorophyll content in barley leaf has widely been studied [11,14,26,28].

Nitrogen uptake and metabolism of flag leaf at the pre-filling stage provide main energy source to grain yield [15]. The photosynthetically active leaf cells of chloroplasts contain most nitrogen [29]. The most of assimilated nitrogen mainly come from photosynthesis. Leaf CO_2_ assimilation rate and nitrogen content per unit area was highly correlated [30]. Depending on physiological status, nitrogen can be stored and assimilated in both leaves and roots [31]. In fully developed leaves, about 75% nitrogen is allocated to chloroplasts, and mostly used for synthesizing components of photosynthetic apparatus [32]. A positive correlation was found between photosynthetic capacity of leaves and their nitrogen content [33]. In past few years, some studies have reported that nitrogen content in leaves was quantitative trait and controlled by multiple genes in barley. Stable QTLs were detected, but phenotypic contribution from each QTL was small [12,15,29].

Plant water status plays an important role in plant growth, development, and keeping yield stability [34]. The physiological and morphological traits such as photosynthesis, transpiration of flag leaves and grain yield are closely correlated with plant water status [35,36]. In water deficit environment, crop must increases water use efficiency to resist drought, and sustains normal growth [37]. Relative water content (RWC) was widely used to measure water status in barley [38]. RWC is an important determinant of leaf metabolic activity, and reflects water balance in tissues [39]. Maintenance of certain level of RWC can increase yield and its stability in cereals [38]. As RWC is related to plant water-status, it can be used to evaluate water level in plant at a specific growth stage. It has been reported that RWC has a positive relationship with yield in cereals [36]. QTLs associated with RWC were detected on chromosome 6H in different water conditions and developmental stages [16,40,41].

In present study, a DH population derived from the cross of Huaai 11 × Huadamai 6 was used to identify QTLs underlying physiological and morphological traits of flag leaf at the pre-filling stage. The identified QTLs can be used for molecular assisted selection (MAS) in barley breeding.

## Results

### Phenotype analysis of the double population and parents

The statistics of 7 physiological and 3 morphological traits of flag leaf at the pre-filling stage were shown in Table 1. The values of Pn, Gs, Ci, Tr, RWC, SPD and LNC in Huaai 11 were higher than those in Huadamai 6. The values of LA, FLL and FLW were higher in Huadamai 6 than those in Huaai 11. The *t*-test showed that two parents were significant difference on all traits (p < 0.05). All traits displayed a normal distribution with the skewness and kurtosis among −1 and 1 (Table 1). Analysis of variance showed that genotype effects were significant (P < 0.01) for all traits studied. Effects between years were not significant (P > 0.05) except Pn, Gs and Tr traits. Genotype × year interactions were significant (P < 0.05) for all traits except LA, FLL and FLW (Table 2). In addition, all 7 physiological and 3 morphological traits at the pre-filling stage showed highly phenotypic variation in the DH population. The variable coefficients ranged from 5.22% to 30.91% in 2012, and 11.50% to 28.50% in 2013. Transgressive segregation in both directions was observed for all traits (Table 1). Heritability (Table 1) ranged from 44.13% to 80.67% and 52.66% to 85.57% in 2012 and 2013, respectively.

**Table 1:** **The statistics of the 122 lines from DH population and parents for the 7 physiological and 3 morphological traits based on data from each year (2012 and 2013)**

**Trait**	**Year**	**Huadamai6**		**Huaai11**		**ST**	**DH lines**							
		**Mean**	**SD**	**Mean**	**SD**		**Max**	**Min**	**Mean**	**SD**	**Skewness**	**Kurtosis**	**CV (%)**	**H (%)**
Pn	2012	26.00 ± 1.17	2.87	29.38 ± 0.55	1.35	0.041^*^	32.56	19.72	25.15 ± 0.24	2.62	0.23	−0.13	10.41	44.13
	2013	22.77 ± 0.10	1.17	25.03 ± 2.71	4.69	0.031^*^	27.77	14.44	20.31 ± 0.21	2.33	0.26	0.39	11.50	56.85
Gs	2012	0.43 ± 0.03	0.06	1.03 ± 0.03	0.07	0.000^**^	1.02	0.21	0.56 ± 0.02	0.17	0.19	−0.53	30.91	53.34
	2013	0.46 ± 0.01	0.01	0.83 ± 0.07	0.12	0.036^*^	0.93	0.20	0.41 ± 0.01	0.14	1.00	1.00	33.05	58.56
Ci	2012	255.83 ± 2.70	6.62	308.67 ± 1.09	2.66	0.000^**^	316.51	197.1	266.84 ± 2.38	26.24	−0.78	0.19	9.84	60.76
	2013	261.32 ± 1.87	3.23	294.89 ± 0.42	0.73	0.005^**^	315.71	216.3	268.01 ± 1.77	19.55	0.14	−0.31	7.29	65.23
Tr	2012	6.45 ± 0.30	0.73	9.28 ± 0.16	0.39	0.001^**^	12.83	4.87	8.21 ± 0.14	1.59	0.04	−0.38	19.32	47.65
	2013	7.47 ± 0.04	0.08	9.86 ± 1.00	1.73	0.028^*^	10.41	3.78	6.27 ± 0.13	1.44	0.38	−0.34	22.95	52.66
LA	2012	27.18 ± 0.88	2.80	12.02 ± 0.83	2.63	0.000^**^	30.42	9.66	17.89 ± 0.37	4.08	0.69	0.19	22.82	78.98
	2013	26.66 ± 1.80	4.76	18.05 ± 1.43	3.80	0.002^**^	37.79	10.37	21.52 ± 0.47	5.18	0.63	0.55	24.10	83.56
FLL	2012	26.62 ± 1.08	3.41	14.36 ± 0.81	2.57	0.000^**^	28.02	13.04	17.94 ± 0.24	2.66	0.28	−0.43	14.84	80.67
	2013	22.31 ± 0.88	2.34	15.84 ± 0.88	2.33	0.000^**^	27.39	12.86	19.09 ± 0.26	2.88	0.44	0.21	15.08	85.57
FLW	2012	2.03 ± 0.13	0.40	1.48 ± 0.05	0.16	0.003^**^	2.20	1.22	1.67 ± 0.02	0.19	0.56	0.27	11.60	69.34
	2013	1.97 ± 0.04	0.11	1.56 ± 0.06	0.15	0.012^*^	2.18	1.21	1.74 ± 0.02	0.18	0.10	0.13	10.53	76.56
RWC	2012	80.96 ± 0.52	1.65	87.13 ± 0.95	3.01	0.015^*^	92.26	73.53	82.68 ± 0.39	4.31	0.11	−0.59	5.22	50.56
	2013	82.62 ± 3.90	8.71	86.05 ± 3.59	8.02	0.050^*^	94.23	72.08	83.38 ± 0.41	4.49	−0.24	−0.16	5.38	57.67
SPD	2012	52.50 ± 1.23	2.13	65.87 ± 0.79	1.37	0.007^**^	71.93	51.17	62.33 ± 0.38	4.17	−0.27	−0.15	6.69	49.56
	2013	51.63 ± 3.17	5.49	62.83 ± 1.79	3.10	0.035^*^	66.33	48.33	59.07 ± 0.33	3.67	−0.48	0.41	6.22	57.89
LNC	2012	2.90 ± 0.07	0.17	4.70 ± 0.25	0.60	0.002^**^	7.88	1.41	4.79 ± 0.12	1.38	−0.39	−0.53	28.76	70.45
	2013	3.84 ± 0.18	0.43	5.01 ± 0.21	0.51	0.000^**^	7.96	1.68	4.89 ± 0.13	1.39	−0.38	−0.38	28.50	62.45

^*, **^: Significant at 0.05, 0.01 level, respectively.

ST: Significant; CV: Coefficient of variation; H: Heritability.

**Table 2:** **Variance analysis of 7 physiological and 3 morphological traits of 122 barley DH lines, sum of squares was shown**

**Source**	**Pn**	**Gs**	**Ci**	**Tr**	**LA**	**FLL**	**FLW**	**RWC**	**SPD**	**LNC**
Genotype	7055.203^**^	16.755^**^	495793.084^**^	1796.972^**^	28542.641^**^	7344.367^**^	31.652^**^	13868.241^**^	14379.196^**^	757.609^**^
Year	3651.228^**^	6.290^*^	289.943	703.441^**^	317.510	161.339	1.813	73.509	603.316	3.493
Genotype × Year	2593.407^**^	6.803^*^	52994.676^*^	646.039^**^	1371.505	671.623	3.679	2100.091^*^	2570.247^*^	96.884^*^

^*, **^: Significant at 0.05 and 0.01 level, respectively.

### Correlation analysis

Correlations among Pn, Gs, Ci, and Tr were significant positive (P < 0.01, Table 3). Three morphological traits, LA, FLL and FLW, were also significantly positive correlated with each other (P < 0.01, Table 3). Significant positive correlation between Pn and SPD was detected with correlation coefficient of 0.335 in 2012 and 0.265 in 2013 (P < 0.01). LNC was significantly correlated with SPD (r = 0.283 in 2012 and 0.381 in 2013, P < 0.01). A negative correlation between Pn and LA was observed with r = −0.515 (year 2012) and −0.225 (year 2013) (P < 0.05). RWC was not significantly (P > 0.05) correlated with other traits except LA in 2013.

**Table 3:** **Correlation analysis among 7 physiological and 3 morphological traits based on data from each year**

**Trait**	**Pn**	**Gs**	**Ci**	**Tr**	**LA**	**FLL**	**FLW**	**RWC**	**SPD**	**LNC**
Pn		0.655^**^	0.474^**^	0.675^**^	−0.515^**^	−0.416^**^	−0.562^**^	0.088	0.335^**^	0.002
Gs	0.657**		0.892^**^	0.918^**^	−0.454^**^	−0.407^**^	−0.450^**^	0.067	0.527^**^	0.160
Ci	0.373**	0.891**		0.767^**^	−0.482^**^	−0.477^**^	−0.422^**^	−0.044	0.499^**^	0.171
Tr	0.701**	0.930**	0.830**		−0.498^**^	−0.422^**^	−0.517^**^	0.055	0.612^**^	0.120
LA	−0.225*	−0.376**	−0.417**	−0.497**		0.864^**^	0.861^**^	0.171	−0.472^**^	−0.082
FLL	−0.188*	−0.390**	−0.428**	−0.504**	0.942**		0.585^**^	0.055	−0.392^**^	−0.025
FLW	−0.213*	−0.336**	−0.390**	−0.440**	0.863**	0.684**		0.165	−0.420^**^	−0.017
RWC	0.017	0.021	0.006	−0.097	0.183*	0.127	0.144		−0.050	0.088
SPD	0.265**	0.193*	0.274**	0.253**	−0.392**	−0.377**	−0.355**	−0.003		0.283^**^
LNC	0.011	0.110	0.201*	0.144	−0.216*	−0.231*	−0.144	−0.011	0.381**	

^*, **^: Significant at 0.05, 0.01 level, respectively.

Values above the diagonal are correlation coefficients in 2012; values below the diagonal are correlation coefficients in 2013.

### QTL analysis

**Figure 1:**
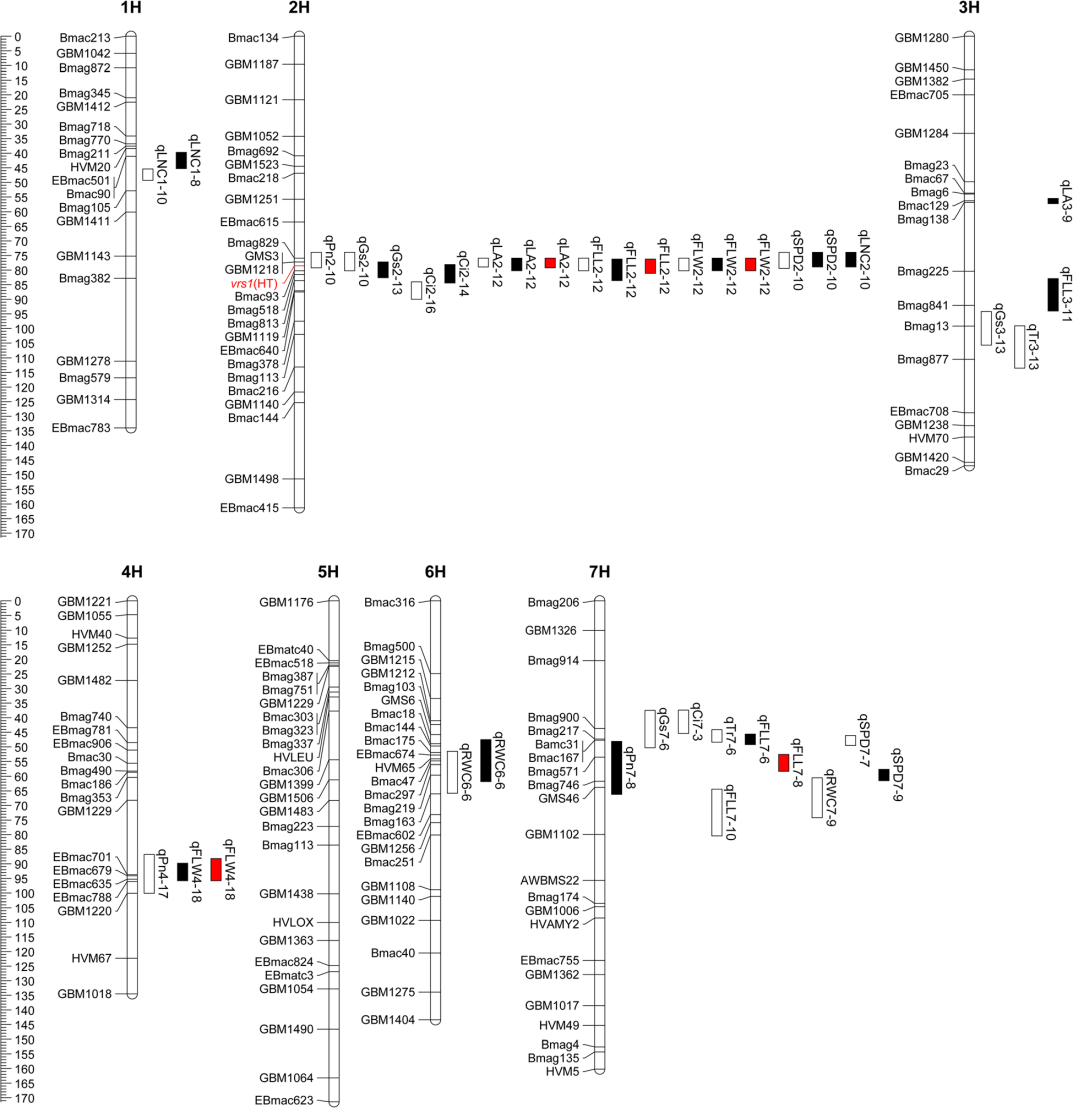
**Chromosome location of QTL associated with 7 physiological (2012, 2013) and 3 morphological traits (2012, 2013 and mean values) detected in the Huaai 11 × Huadamai 6 DH population.** Genetic distance scales in centiMorgans (*cM*) are placed at left margin. Location of QTL is indicated for year 2012 (*white bar*), year 2013 (*black bar*) and mean values (*red bar*). The head type trait was shown on linkage map (*red marker*).

A total of 38 QTLs for 7 physiological and 3 morphological traits were detected and mapped (Figure 1; Table 4). 18 and 15 QTLs were detected in 2012 and 2013, respectively. Five QTLs based on mean value of each trait were detected for LA, FLL and FLW. The detected QTLs accounted for 7.14% - 24.58% and 6.53% - 25.36% phenotypic variation in 2012 and 2013, respectively. The QTLs based on mean values of LA, FLL and FLW explained 14.23% - 31.29% phenotypic variation.

**Table 4:** **QTL detected for 7 physiological and 3 morphological traits based on data form year 2012, 2013 and mean value form two years**

**Trait**	**Year**	**QTL**	**Chromosome**	**Nearest marker**	**Position (cM)**	**Interval (cM)**	**LOD**	**Explained variance (%)**	**Additive effect**
Pn	2012	qPn2-10	2	Bmag829	75.9	73.9 - 79.2	3.43	8.66	−0.94
	2012	qPn4-17	4	EBmac788	96.1	86.8 - 100.1	4.64	12.63	1.09
	2013	qPn7-8	7	Bmag571	53.5	48.1 - 66.2	4.86	13.56	−1.19
Gs	2012	qGs2-10	2	Bmag829	75.9	73.9 - 80.2	3.49	7.78	−0.05
	2012	qGs3-13	3	Bmag13	97.6	94.2 - 105.7	5.44	12.58	−0.07
	2012	qGs7-6	7	Bmac31	47.1	37.5 - 50.3	5.95	13.92	−0.07
	2013	qGs2-13	2	Bmac93	80.2	77.2 - 82.6	3.04	7.47	−0.04
Ci	2012	qCi2-16	2	GBM1119	87.1	84.0 - 90.0	4.93	13.75	−9.62
	2012	qCi7-3	7	Bmag914	42.4	37.4 - 45.4	5.01	13.98	−9.78
	2013	qCi2-14	2	Bmag518	81.5	78.1 - 84.4	3.75	10.69	−7.78
Tr	2012	qTr3-13	3	Bmag13	103.6	99.1 - 113.5	4.51	14.00	−0.69
	2012	qTr7-6	7	Bmac31	47.1	44.1 - 48.4	5.58	14.02	−0.71
LA	2012	qLA2-12	2	GBM1218	77.2	75.9 - 78.9	7.09	18.80	2.17
	2013	qLA2-12	2	GBM1218	77.2	75.9 - 80.2	5.22	12.48	2.00
	2013	qLA3-9	3	Bmac129	56.3	55.5 - 57.3	3.77	8.72	−1.81
	Mean	qLA2-12	2	GBM1218	77.2	75.9 - 79.2	14.17	29.83	2.53
FLL	2012	qFLL2-12	2	GBM1218	77.2	75.9 - 80.2	9.98	24.58	1.52
	2012	qFLL7-10	7	GMS46	72.4	64.4 - 80.4	4.59	13.04	1.06
	2013	qFLL2-12	2	GBM1218	79.2	76.2 - 83.6	10.16	25.36	1.53
	2013	qFLL3-11	3	Bmag225	83.4	82.9 - 94.0	3.53	9.76	0.98
	2013	qFLL7-6	7	Bmac31	47.1	45.6 - 49.2	3.22	7.07	0.79
	Mean	qFLL2-12	2	GBM1218	79.2	76.2 - 81.1	14.98	31.29	1.55
	Mean	qFLL7-8	7	Bmag571	55.5	52.6 - 58.3	8.81	16.66	1.14
FLW	2012	qFLW2-12	2	GBM1218	77.2	75.9 - 80.2	5.57	13.63	0.08
	2013	qFLW2-12	2	GBM1218	77.2	75.9 - 80.2	7.86	20.93	0.09
	2013	qFLW4-18	4	GBM1220	93.8	89.8 - 95.8	3.19	7.11	−0.05
	Mean	qFLW2-12	2	GBM1218	77.2	75.9 - 80.2	7.31	14.23	0.08
	Mean	qFLW4-18	4	GBM1220	93.8	88.2 - 95.8	8.90	22.06	−0.09
RWC	2012	qRWC6-6	6	GMS6	57.8	51.5 - 65.8	6.35	21.43	2.03
	2012	qRWC7-9	7	Bmag746	62.3	60.5 - 74.2	5.77	15.31	−1.74
	2013	qRWC6-6	6	GMS6	53.8	47.5 - 61.8	3.15	11.76	1.72
SPD	2012	qSPD2-10	2	Bmag829	75.9	73.9 - 79.4	6.97	17.28	−2.08
	2012	qSPD7-7	7	Bmac167	47.5	46.1 - 49.5	4.57	10.78	−1.57
	2013	qSPD2-10	2	Bmag829	75.9	73.9 - 78.9	6.27	15.44	−1.56
	2013	qSPD7-9	7	Bmag746	58.9	57.6 - 61.5	4.48	10.64	−1.34
LNC	2012	qLNC1-10	1	EBmac501	48.1	45.4 - 49.3	3.27	7.14	−0.24
	2013	qLNC1-8	1	Bmag211	41.1	39.7 - 45.3	3.37	8.46	−0.29
	2013	qLNC2-10	2	Bmag829	75.9	73.9 - 78.9	3.12	6.53	−0.17

### Net photosynthetic rate

Three QTL underlying Pn trait were detected. Two QTLs, qPn2-10 and qPn4-17, were detected on chromosome 2H and 4H in 2012. They accounted for 8.66% and 12.63% total phenotypic variation, respectively. The QTL, qPn7-8 on chromosome 7H was detected in 2013, and accounted for 13.56% total phenotypic variation. Both qPn2-10 and qPn7-8 QTLs have alleles from Huaai 11 to increase net photosynthetic rate, the QTL qPn4-17 has allele from Huadamai 6 to increase net photosynthetic rate (Figure 1; Table 4).

### Stomatal conductance

Four QTLs associated with Gs trait were detected. Of them, three QTLs, qGs2-10, qGs3-13 and qGs7-6, were detected in 2012 and mapped on chromosome 2H, 3H and 7H, and accounted for 7.78%, 12.58% and 13.92% total phenotypic variation, respectively. In 2013, one QTL qGs2-13 was detected on chromosome 2H, and accounted for 7.47% total phenotypic variation. All these QTLs have alleles from Huaai 11 to increase stomatal conductance, their values ranged from 0.04 to 0.07 (Figure 1; Table 4).

### Intercellular CO_2_ concentration

Three QTLs for Ci trait were detected. Of them, two QTLs, qCi2-16 and qCi7-3, were mapped on chromosome 2H and 7H in 2012, and accounted for 13.75% and 13.98% total phenotypic variation, respectively. One QTL qCi2-14 was identified in 2013, and accounted for 10.69% total phenotypic variation. These QTLs have alleles from Huaai 11 to increase intercellular CO_2_ concentration (Figure 1; Table 4).

### Transpiration rate

Two QTLs underlying Tr trait were identified in 2012. The QTL qTr3-13 and qTr7-6 accounted for 14.00% and 14.02% total phenotypic variation, respectively. The additive effects of the two QTLs were 0.69 and 0.71, respectively, indicating that the alleles from Huaai 11 increased transpiration rate (Figure 1; Table 4).

### Flag leaf area

Four QTLs underlying LA trait were detected on chromosome 2H and 3H. The QTL, qLA2-12 close to the marker GBM1218, was detected in both years and mean value, and accounted for 18.80% (year 2012), 12.48% (year 2013) and 29.83% (mean value from two years) phenotypic variation. The alleles from Huadamai 6 increased flag leaf area. Another QTL qLA3-9 detected in 2013 accounted for 8.72% phenotypic variation. The allele of QTL qLA3-9 from Huaai 11 increased flag leaf area (Figure 1; Table 4).

### Flag leaf length

Seven QTLs associated with FLL trait were detected. The QTL, qFLL2-12 close to the marker GBM1218 on chromosome 2H, was detected in both years and mean value, and accounted for 24.58% (year 2012), 25.36% (year 2013) and 31.29% (mean value from two years) phenotypic variation. The alleles of the QTL, which increased flag leaf length, came from Huadamai 6. Other four QTLs, qFLL7-10, qFLL3-11, qFLL7-6 and qFLL7-8, accounted for 13.04%, 9.76%, 7.07% and 16.66% total phenotypic variation, respectively. The positive alleles of QTL qFLL7-10, qFLL3-11, qFLL7-6 and qFLL7-8 from Huadamai 6 contributed to the increase in flag leaf length by 1.06, 0.98, 0.79 and 1.14, respectively (Figure 1; Table 4).

### Flag leaf width

For FLW trait, five putative QTLs were identified. The QTL, qFLW2-12 close to the marker GBM1218 on chromosome 2H, was detected in both years and mean value, and accounted for 13.63% (year 2012), 20.93% (year 2013) and 14.23% (mean value from two years) total phenotypic variation. The positive alleles of QTL qFLW2-12 from Huadamai 6 increased flag leaf width. Another QTL qFLW4-18 detected in 2013 and mean value was located on chromosome 4H, and accounted for 7.11% and 22.06% total phenotypic variation, respectively. The alleles of qFLW4-18 from Huaai 11 contributed to the increase in flag leaf width (Figure 1; Table 4).

### Relative water content

Three QTLs underlying RWC were found. The QTL qRWC6-6 nearby the marker GMS6 on chromosome 6H was detected in both years, and accounted for 21.43% (year 2012) and 11.76% (year 2013) phenotypic variation. Their alleles from Huadamai 6 increased relative water content. Another QTL, qRWC7-9 was detected in year 2012 and mapped on chromosome 7H, which accounted for 15.31% phenotypic variation. The allele from Huaai 11 increased relative water content (Figure 1; Table 4).

### Relative chlorophyll content

Four QTLs underlying SPD trait were found. The QTL qSPD2-10 was detected in both years and close to the marker Bmag829 on chromosome 2H, and accounted for 17.28% (year 2012) and 15.44% (year 2013) total phenotypic variation. Two QTLs, qSPD7-7 and qSPD7-9, were mapped on chromosome 7H and close to the marker Bmac167 (year 2012) and Bmag746 (year 2013). They accounted for 10.78% and 10.64% total phenotypic variation in year 2012 and 2013, respectively. All these QTLs have alleles from Huaai 11 contributed to the increase in relative chlorophyll content (Figure 1; Table 4).

### Total nitrogen content

Three QTLs associated with LNC trait were detected. Of them, one QTL, qLNC1-10 on chromosome 1H, was detected in 2012 and accounted for 7.14% phenotypic variation. Two QTLs qLNC1-8 and qLNC2-10 were mapped on chromosome 1H and 2H in 2013, and accounted for 8.46% and 6.53% phenotypic variation, respectively. All these QTLs have alleles from Huaai 11 contributed to the increase in total nitrogen content (Figure 1; Table 4).

## Discussion

QTL analysis is a useful approach to discover and identify favorable alleles in barley [42]. Ren et al. [43] have studied the correlation and QTL of agronomic and quality traits associated with grain yield in a barley DH population. However, QTL associated with physiological and morphological traits of flag leaf at the pre-filling stage have not been systematically analyzed.

Leaf net photosynthetic rate was easily affected by environment factors. It was reported the net photosynthetic rate was different in different environments including illumination intensity, temperature, content of CO_2_ and moisture in the air [44]. In our experiment, we selected 9:00–11:00 am and 2:00–4:00 pm to measure photosynthesis based on the daily change rule of photosynthesis and our operational experience that photosynthesis was stable at these two time periods. In plant developmental stage, the four traits Pn, Gs, Ci and Tr index reflect plant photosynthetic capacity. The all four traits were closely related to grain yield. QTLs underlying Pn, Gs and Tr have been analyzed in rice [2]. Wójcik-Jagła et al. [18] analyzed QTL underlying net photosynthetic rate in barley, and found one QTL nearby the marker bPb-8013 on chromosome 4H in the Suweren × MOB12055 population, one QTL on chromosome 5H in the STH754 × STH836 population. In our study, we detected one QTL nearby the marker EBmac788 on chromosome 4H. The consensus map of Wenzl et al. [20] showed that the marker bPb-8013 is far from EBmac788, indicating that the qPn4-17 was a new QTL identified here. In rice, QTL analysis of several physiological traits related to photosynthesis had been performed [2]. In our study, 9 QTLs controlling Gs, Ci and Tr traits in barley flag leaf were detected. The identified QTLs may be useful for MAS in barley breeding.

To sustain crop growth and development, crop must produce abundant nutrition. The amount of nutrition produced mainly depends on flag leaf associated with Pn, SPD, LNC and LA, which were closely related to grain yield and biomass [3,7,9]. Four QTLs associated with relative chlorophyll content were detected. QTL qSPD2-10 was detected at 75.9 cM in 2012 and 2013, indicating this QTL was stable and less affected by environments. In barley, This et al. [17] detected 12 QTLs underlying chlorophyll content on chromosome 2H, 4H, 5H, 6H and 7H. Xue et al. [11] detected two QTLs underlying chlorophyll content on chromosome 2H. One QTL related to SPD trait has mapped on chromosome 2H [26]. The high density consensus map [42] indicated the qSPD2-10 was close to the QTL (qFC2.2) [11], between marker Bmag0518 and Bmac0093. The QTL qSPD7-7 and qSPD7-9 were close to the centromere of chromosome 7H, and different from the QTL on chromosome 7H reported previously [17,28]. Five QTLs controlling nitrogen content of flag leaf were detected on chromosome 2H, 3H, 5H and 7H [12]. Mickelson et al. [15] detected 19 QTLs on chromosome 3H, 4H, 5H, 6H and 7H associated with nitrogen concentration in flag leaf. Three QTLs underlying LNC trait were detected on chromosome 1H and 2H in our study, indicating that the two QTLs on chromosome 1H may be new QTL underlying nitrogen concentration in flag leaf. The QTL qLNC2-10 on centromere region of chromosome 2H is different from the QTL on chromosome 2H reported previously [12]. Four QTLs associated with flag leaf area were identified. The QTL qLA2-12 on chromosome 2H located at 77.2 cM was detected in both years and mean value. Previous studies reported QTL underlying leaf area on chromosome 1H, 2H, 3H, 4H, 5H and 7H [12,45]. The qLA2-12 on 2HL is different from the QTL reported on 2HS [12]. In our study, one region on chromosome 2H flanked by Bmag829 and GBM1218 contained the qPn2-10, qLA2-12, qSPD2-10 and qLNC2-10 (Figure 1), suggesting that there might be QTL cluster for controlling grain yield on chromosome 2H, and these molecular makers can be used for MAS to improve breeding efficiency.

Since year effects and genotype × year interactions were not significant (p > 0.05) for three morphological traits (LA, FLL, FLW), QTL analysis was performed for data from each year and mean value of two years. In our study, 16 QTLs associated with the 3 morphological traits (LA, FLL and FLW) were identified in two years and mean values, which located on chromosome 2H, 3H, 4H and 7H, respectively. Elberse et al. [46] detected 6 QTLs underlying leaf length on chromosome 1H, 2H, 4H and 5H, 3 QTLs controlling leaf width on chromosome 2H, 4H and 6H. Li et al. [45] reported a chromosome region on 3HS underlying leaf length and leaf area. Gyenis et al. [10] reported 3 QTLs controlling flag leaf length on chromosome 3H, 5H and 7H, and 3 QTLs underlying flag leaf width on 2H, 4H and 5H. Xue et al. [11] detected 2 QTLs controlling flag leaf length on chromosome 5H and 7H, and 2 QTLs controlling flag leaf width on chromosome 5H. The QTL qFLL2-12 located on chromosome 2HL, and is different from the QTL reported on 2HS [46]. The QTL, qFLW2-12 located on chromosome 2HL, and is different from those QTLs reported on 2HS [10,46]. The 3 morphological traits were significantly correlated with each other (Table 3), a common QTL close to the marker GBM1218 on chromosome 2H controlled these traits (Figure 1; Table 4). Phenotypic correlations among traits and identification of QTL were generally in good agreement. QTLs controlling LA, FLL and FLW were detected on the same region of chromosome 2H in both years and mean values. This region was close to the marker GBM1218, and contained the qLA2-12, qFLL2-12 and qFLW2-12 (Figure 1), indicating that this region is important for controlling morphological trait in barley. Moreover, all QTL positive alleles except qLA3-9 and qFLW4-18 were contributed by Huadamai 6.

Photosynthesis process assimilates H_2_O and CO_2_ to produce carbohydrates, and can be influenced by plant water status. Relative water content of flag leaf is one important assessment criterion about plant water status [47]. In our study, one common QTL on the chromosome 6H is close to marker GMS6. Teulat et al. [40] detected one QTL on the chromosome 6H under two different water treatments. Another study also detected two QTLs on the long arm of chromosome 6H [16]. Previous studies on QTL underlying RWC trait of barley flag leaf found 2 genome regions on the chromosome 6H associated with RWC, which were close to BCD348B and BCD1, respectively [13,16,40,41]. These suggested that there might be a QTL cluster in this region. Chromosome 7H have 3 genome regions associated with RWC, which are nearby RZ123, Acl3 and Bass1B, respectively [13,16,40,41]. The QTL qRWC6-6 detected in present study was close to the marker BCD348B, and the QTL qRWC7-9 was close to the marker RZ123.

In our study, two QTL cluster regions associated with physiological and morphological traits, one each on the chromosome 2H and 7H, were observed (Figure 1). The head type trait was mapped on chromosome 2H between marker GBM1218 and Bmac93, which is close to the QTL cluster region (Figure 1). The heading date trait was also mapped on chromosome 2H close to marker GBM1218 in the QTL cluster region [43]. The dwarfing gene was mapped on chromosome 7H in the QTL cluster region [48]. The head type, heading date and plant height traits were considered to be significantly associated with grain yield [43,49,50]. The *vrs1* locus controlling head type was mapped on chromosome 2H [51,52]. From http://wheat.pw.usda.gov/GG2/index.shtml, we found that the marker GBM1218 was close to *vrs1* locus. Considering all information here, we suggested that the head type, heading date and plant height traits might be highly associated with these physiological and morphological traits, and could be considered as important factors to control grain yield. Pleiotropy and linkage were present in some important traits associated with yield parameters [53]. In present study, there exist widely co-localized QTL between physiological and morphological traits, such as Pn, Gs, SPD, LNC traits on chromosome 2H nearby the marker Bmag829, and LA, FLL, FLW traits on chromosome 2H nearby the marker GBM1218, where the *vrs1* locus was mapped to. There is always a concentration of QTL effects in the *vrs1* locus. The co-localization of these QTL is most likely due to pleiotropic effect or gene linkage. Distinguishing linkage from pleiotropy is important for breeding purposes, especially if both desirable and undesirable traits are associated with the same locus or QTL region [13]. Thus, in order to distinguish linkage and pleiotropy, further study is needed.

## Conclusions

In this study, physiological and morphological traits showed significant difference in two parents Huaai 11 and Huadamai 6. We found that chromosome 2H and 7H each contained a QTL cluster region controlling grain yield. The molecular makers (Bmag829 and GBM1218) identified here can be used for marker assisted selection to improve breeding efficiency.

## Methods

### Plant materials and field experiments

A barley DH population consisting of 122 DH lines was derived from a cross between dwarfing barley cultivar Huaai 11 (six-rowed and dwarfing) and common feed barley cultivar Huadamai 6 (two-rowed and tall plant) using anther culture. The two parents Huaai 11 and Huadamai 6 are significant difference in plant height [48], physiological and morphological traits of flag leaf. Experiment was conducted in a rain shelter of the Huazhong Agricultural University, Wuhan, China. Side window of the rain shelter was open to make inside temperature and radiation similar to outside condition. The experiments were performed in year 2012 and 2013. The DH lines and parents were grown in a plot of 1.5 m long with interval of 0.6 m and 3 replications using a randomized complete block design. Twenty seeds from each DH line and parent were sown in two rows per plot. Prior to seeding, compound fertilizer (60 g/m^2^) was applied, and 20 g/m^2^ of urea were applied at the elongation stage. At the pre-filling stage, fully expanded flag leaves from main spike were sampled and used to measure 7 physiological and 3 morphological traits.

### Quantification of physiological traits of flag leaf at the pre-filling stage

Four physiological traits, net photosynthetic rate (Pn, umol CO_2_ m^−2^ s^−1^), stomatal conductance (Gs, mol H_2_O m^−2^ s^−1^), intercellular CO_2_ concentration (Ci, μmol CO_2_ mol^−1^) and transpiration rate (Tr, mmol H_2_O m^−2^ s^−1^), were measured using LI6400 XT Portable photosynthesis system according to the methods described in [54]. Measuring time was selected during 9:00–11:00 am and 2:00–4:00 pm. Three fully expanded and sun-exposed topmost flag leaves on main stem from each replication were measured. The parameters were set as follow: LeafFan at Fast, Flow at 500 μmols^−1^, Mixer at 400 ppm, Temp at off and Lamp according to the light intensity. The data was recorded after these parameters reading became relatively stable (usually about 1 min).

### RWC quantification

Weighing method was applied to measure relative water content (RWC) in flag leaves [16]. A flag leaf was sampled from each replication and measured 3 times. After fresh leaves weighted (fw), leaves were immersed in a sealed bag containing distilled water, and kept for 24 hours to achieve completely rehydration. Then the turgid leaves were weighted (tw), and dried to constant weight (dw). RWC was calculated as: RWC = (fw-dw)/(tw-dw) × 100%.

### SPD quantification

SPAD-502 chlorophyll photometer was used to measure relative chlorophyll content (SPD) of flag leaves at the pre-filling stage. Four flag leaves from each replication were measured. SPD values in the top, medium and bottom part of flag leaf were averaged from three replications.

### LNC quantification

Leaf nitrogen concentration (LNC) was measured using the Kjeldahl Nitrogen determination method. Ten flag leaves from each replication were collected at the pre-filling stage, immediately dried at 105°C in an oven for at least 4 h and then ground into powder using Whirlwind grinding JFS-13A, and stored at 80°C until use. Hanon SH220 was used to digest 0.2 g flag leaf powder. The digestive juice was put in distillation Hanon K9840 Kjeldahl Auto Analyzer to measure consumed volume of standard HCL. Total nitrogen in flag leaf (%) was calculated using the formula:

$$ LNC\left(\%\right)=\frac{C\times \left(V-V0\right)\times 14\times 100}{M\times 10\times 1000}\times 100 $$

Where: *C* is concentration of standard HCL in the titration (mol/L); *V* is consumed volume of standard HCL in the titration sample (ml); *V*_*0*_ is consumed volume of standard HCL in the titration blank group (ml); 14 is the atomic mass of nitrogen (g); 100 is total volume of digestive juice (ml); 10 is extract volume of digestive juice (ml); *M* is powder weight of sample (g).

### Quantification of morphological traits

Flag leaf area (LA, area of total leaf, in cm^2^), flag leaf length (FLL, from base of ligula to tip of leaf, in cm) and flag leaf width (FLW, widest part of leaf, in cm) were measured using LI-3000C Portable Area Meter. Four flag leaves of main spike from each replication were measured.

### Data analysis

Statistics, correlation and QTL analyses were performed for the data from each year. Mean value from two years was also used for QTL analysis if genotype × year interaction did not reach significant level for that trait. Homogeneity of variance and normality of distribution were tested before analysis of variance (ANOVA). Heritability was calculated for each trait using ANOVA analysis. The General Linear Model was used for analysis of variance. All analyses were performed using IBM SPSS Statistics 19 software. P value less than 0.05 was considered as significance.

Linkage map was constructed using the software MAPMAKER version 3.0 [55]. Genetic distance (centiMorgans, *cM*) was derived from Kosambi function. The software MapChart 2.2 was used to draw QTL location on the map.

Total of 153 SSR markers evenly distributed on 7 barley chromosomes were used to construct a barley linkage map as previous described [43,48]. The most likely location of QTL and their genetic effects were detected by composite interval mapping (CIM) using QTL Cartographer version 2.5 [56]. After performing 1000 permutation test, a LOD threshold of 3.0 was used to declare presence of a putative QTL in a given genomic region [57]. Composite interval mapping (CIM) was employed to identify QTL using Model 6 of the Standard module. Cofactors were chosen using the forward-backward method of stepwise regression. The genome was scanned at 2 cM intervals and the window size set at 10 cM. Percentage of phenotypic variation explained and additive effect of each QTL were also calculated by QTL Cartographer 2.5. QTL name was composed of q, the abbreviation of trait, the location of chromosome and the marker position on chromosome.

## Abbreviations

DH: Double haploid
QTL: Quantitative trait locus
MAS: Marker assisted selection
Pn: Net photosynthetic rate
Gs: Stomatal conductance
Ci: Intercellular CO_2_ concentration
Tr: Transpiration rate
LA: Flag leaf area
FLL: Flag leaf length
FLW: Flag leaf width
RWC: Relative water content
SPD: Relative chlorophyll content
LNC: Leaf nitrogen concentration
